# Effect of various Danshen injections on patients with coronary heart disease after percutaneous coronary intervention: A protocol for a systematic review and network meta-analysis

**DOI:** 10.1097/MD.0000000000011062

**Published:** 2018-06-15

**Authors:** Zehao Zhu, Yuanping Wang, Weilin Liao, Huimin Li, Dawei Wang

**Affiliations:** a The Second Clinical College of Guangzhou University of Chinese Medicine, Guangzhou; b Shunde Hospital Affiliated of Guangzhou University of Chinese Medicine, Shunde, China.

**Keywords:** coronary heart disease, Danshen injection, percutaneous coronary intervention, protocol, systematic review

## Abstract

**Background:**

Patients with coronary heart disease (CHD) who undergo percutaneous coronary intervention (PCI) have a certain risk of vascular complications, including coronary restenosis and thrombosis. Many recent randomized controlled trials have reported that Danshen injection (DSI) combined with conventional Western medicine can significantly reduce the occurrence of major cardiovascular adverse events in patients with CHD after PCI. However, there are many types of DSIs, and no study has yet compared each type. Therefore, we propose a study protocol for the systematic evaluation of the efficacy of various DSIs in the treatment of CHD after PCI.

**Methods:**

We will search the following electronic databases for randomized controlled trials evaluating the effect of DSI in patients with CHD after PCI: PubMed, Embase, Web of Science, Cochrane Library, Scopus, Ovid Evidence-Based Medicine Reviews, China National Knowledge Infrastructure, and Chinese Biomedicine Literature Database. Each database will be searched from inception to April 2018. The entire process will include study selection, data extraction, risk of bias assessment, pairwise meta-analyses, and network meta-analyses.

**Results:**

This proposed study will compare the efficacy of different DSIs in the treatment of patients with CHD after PCI. The outcomes will include major cardiovascular adverse events and left ventricular ejection fraction.

**Conclusion:**

This proposed systematic review will evaluate the different advantages of various types of DSIs in the treatment of patients with CHD after PCI.

**Registration:**

PROSPERO (registration number: CRD42018092705).

## 1 Introduction

Coronary heart disease (CHD) is a serious cardiovascular disease that causes myocardial ischemia, hypoxia, and even necrosis.^[1]^ CHD is more common in developed countries in Europe and America than in undeveloped countries. Each year, it is estimated that approximately 660,000 people in the United States have a new coronary attack.^[2]^ The mortality rate of CHD is the highest of all cardiovascular disease, accounting for about 40% of all cardiovascular disease-related deaths.^[3]^ With the aging of the global population, CHD has become a major public health problem that seriously threatens human life and health.^[4]^

Currently, the main treatment for CHD is percutaneous coronary intervention (PCI). PCI not only relieves palpitations and angina pectoris, but also reduces the mortality of CHD.^[5]^ However, PCI carries a varying degree of risk of in-stent restenosis and thrombosis, even in patients treated with anticoagulation, anti-aggregation, lipid-regulating, vasodilating, and antihypertensive drugs. The incidence of these post-PCI complications is 20% to 30%.^[6]^ Conventional treatment drugs used to prevent these complications result in many adverse reactions. For example, statins can induce abnormal transaminases, myositis, and even rhabdomyolysis,^[7]^ anticoagulants increase the risk of bleeding from the skin, mucous membranes, and gastrointestinal tract,^[8]^ and angiotensin converting enzyme inhibitors (ACEI) can cause decreased renal function and angioedema.^[9]^ These adverse reactions limit the use of conventional drugs after PCI. Therefore, it is very important to identify effective and therapeutic drugs with minimal side effects to prevent in-stent restenosis and thrombosis after PCI.

In recent years, Danshen injection (DSI) has been increasingly widely used in cardiovascular diseases, especially in Chinese medical units. The main ingredient of DSI is *Salvia miltiorrhiza* extract, including tanshinone and salvianolic acid A. Animal experimental studies have shown that tanshinone inhibits calcium influx in rat vascular smooth muscle cells and relaxes coronary arteries, thereby improving blood flow.^[10]^ In addition, salvianolic acid A increases mitochondrial membrane potential, protects cardiomyocytes from hypoxic injury in rats, and simultaneously causes antiplatelet aggregation and anti-thrombosis effects.^[11,12]^ Clinical studies have shown that, compared with Western medicine alone, DSI combined with conventional Western medicine can reduce the incidence of major cardiovascular adverse events in patients with CHD after PCI.^[13,14]^ However, there are many types of DSIs, and it is not clear which type is superior. The present proposed study will use network meta-analysis (NMA) to systematically evaluate the efficacy and safety of various types of DSI in the treatment of patients with CHD after PCI, providing a reference for clinical practice.

## 2 Methods

### 2.1 Inclusion criteria for study selection

#### 2.1.1 Types of included studies

The proposed study will include randomized controlled trials (RCTs) that have evaluated all types of DSIs for the treatment of patients with CHD undergoing PCI. There will be no restrictions on the geographical area, time, and/or language of publication.

#### 2.1.2 Participants

All patients must meet the diagnostic criteria for adult CHD established by the American College of Cardiology/American Heart Association,^[15]^ and all patients must have undergone PCI. There will be no limits placed on basic patient characteristics (including region, race, and sex).

#### 2.1.3 Interventions

The experimental group must have been treated with DSI or DSI combined with conventional Western medicine. The control group must have been treated with different types of DSI, or different types of DSI combined with conventional Western medicine, or only conventional Western medicine.

#### 2.1.4 Outcomes

##### 2.1.4.1 Primary outcomes

Major cardiovascular adverse events, including myocardial infarction, re-PCI, and coronary bypass reconstruction.

##### 2.1.4.2 Secondary outcomes

Left ventricular ejection fraction, blood lipid changes, coagulation function changes, cardiac enzyme changes, and inflammatory factor levels.

### 2.2 Sources of data

The study search will be mainly based on electronic databases, including PubMed, Embase, Web of Science, Cochrane Library, Scopus, Ovid Evidence-Based Medicine Reviews, China National Knowledge Infrastructure, and Chinese Biomedicine Literature Database. The following search strategy will be used in PubMed:

#1 “coronary heart disease”[MeSH Terms] OR “coronary heart disease”[Title/Abstract] OR “coronary atherosclerotic heart disease”[Title/Abstract] OR “coronary”[Title/Abstract] OR “angina pectoris”[Title/Abstract] OR “angina”[Title/Abstract] OR “pectoris”[Title/Abstract] OR “myocardial infarction”[Title/Abstract] OR “acute myocardial infarction”[Title/Abstract] OR “infarction”[Title/Abstract] OR “myocardial ischemia”[Title/Abstract] OR “myocardial ischemic”[Title/Abstract] OR “ischemic heart failure”[Title/Abstract]
#2 “percutaneous coronary intervention”[MeSH Terms] OR “percutaneous coronary
intervention”[Title/Abstract] OR “percutaneous coronary artery intervention”[Title/Abstract] OR “PCI”[Title/Abstract]
#3 “Danshen injection”[Title/Abstract] OR “Danshen chuanxiongqin injection”[Title/Abstract] OR “Danshen glucose injection”[Title/Abstract] OR “Danhong injection”[Title/Abstract]
OR “Danxiangguanxin injection”[Title/Abstract] OR “Xiangdan injection”[Title/Abstract]
OR “*Salvia* injection”[Title/Abstract] OR “*Salvia miltiorrhiza*”[Title/Abstract] OR
“Salvianolate injection”[Title/Abstract] OR “Salvianolate lyophilized injection”[Title/Abstract] OR “Compound *Salvia* injection”[Title/Abstract] OR “Guanxinning injection”[Title/Abstract] OR “Tanshinone IIA sulfonate injection”[Title/Abstract]
#4 #1 AND #2 AND #3

### 2.3 Data extraction and risk of bias assessment

#### 2.3.1 Study selection

The initial selection will involve scanning of the titles and abstracts of the retrieved studies. The full text of relevant studies will then be reviewed for study inclusion in accordance with the inclusion criteria.

#### 2.3.2 Data extraction

Two staff members will perform data extraction. The extracted data will include region, ethnicity, male/female ratio, age range, disease type, drug dosage, and outcome indicators. If there is disagreement between these 2 staff members regarding the extracted data, the matter will be resolved through discussion or will be submitted to a third staff member for adjudication.

#### 2.3.3 Risk of bias assessment

We will assess the risk of bias in each study using the Jadad scale. The assessment includes the following 6 aspects: sequence generation, allocation concealment, blinding, incomplete outcome data, selective outcome reporting, and other bias.^[16]^

### 2.4 Statistical analysis

#### 2.4.1 Network plot

The included studies will be linked through a network plot consisting of dots and lines. The dots will represent each intervention, and the lines will represent direct comparisons between different interventions. The size of the dot will indicate the number of participants (with a larger dot indicating a relatively greater number of participants), while the thickness of the line will indicate the number of studies (with a thicker line indicating a relatively greater number of studies).

#### 2.4.2 Pairwise meta-analyses

Pairwise meta-analyses will be performed using Stata 13.0 software. The effect size of dichotomous variables (major cardiovascular adverse events) will be assessed using the odds ratio, while the effect size of continuous variables (left ventricular ejection fraction, blood lipid changes, coagulation function changes, cardiac enzyme changes, and inflammatory factor levels) will be assessed using the mean difference. The confidence intervals (CI) will be set to 95%. When the heterogeneity test shows *I*^2^ < 30% or *P* > .05, the fixed effect model will be selected. When the heterogeneity test indicates *I*^2^ ≥ 30% or *P* ≤ .05, the random effect model will be selected, and we will investigate the source of the heterogeneity through sensitivity and subgroup analyses. Funnel charts will be used to evaluate the presence of a publication bias.

#### 2.4.3 Network meta-analysis

A NMA will be performed using WinBUGS 14 (MRC Biostatistics Unit, Cambridge University, UK).^[17]^ The effect sizes of dichotomous and continuous variables will be consistent with the pairwise meta-analysis. A potential scale reduction parameter of close to 1 indicates that the convergence is better and the result is credible. If the 95% CI of the odds ratio includes 1, or the 95% CI of the mean difference contains 0, this indicates that there is no significant difference between interventions. Ultimately, all interventions will be ranked based on the Markov Chain Monte Carlo theory.

#### 2.4.4 Inconsistency testing

When the “Inconsistency Factors” value is close to 0 and its 95% CI crosses 0, this indicates that the degree of inconsistency is low, and the consistency model will be selected. Conversely, when the “Inconsistency Factors” value is far from 0 and its 95% CI does not contain 0, the degree of inconsistency is larger, and the inconsistency model will be selected.

#### 2.4.5 Node split analysis

When the inconsistency test indicates a large degree of inconsistency, further node split analysis will be performed. If *P* < .05, this indicates that the results of direct and indirect comparisons are inconsistent. If this situation arises, we will conduct in-depth analysis based on the principles of homogeneity and transmissibility, and look for the sources of inconsistency.

### 2.5 Ethics and dissemination

The data used in this systematic review will be collected from published studies. Based on this, the study does not require ethical approval. The results of this study will eventually be published in a peer-reviewed journal.

## 3 Discussion

CHD is a serious cardiovascular disease that is the most common cause of death in developed countries.^[18,19]^ As PCI can reduce CHD mortality and relieve angina pectoris, it has attracted attention as an effective long-term treatment. However, even in patients who are treated after PCI with anticoagulation, anti-aggregation, lipid-regulating, and other conventional treatments, there are varying degrees of risk of coronary restenosis and thrombosis.^[20]^ Therefore, the search for effective treatment to prevent complications after PCI has become a research subject of great interest. Recent studies have shown that *S miltiorrhiza* can prevent endothelial injury, reduce the release of inflammatory factors, and prevent platelet aggregation.^[21–23]^ Furthermore, DSI combined with Western medicine effectively reduces the incidence of adverse events after PCI.^[24]^ However, there are a wide variety of DSIs available, and no NMA has shown which kind of DSI is most effective. Therefore, we aim to perform a NMA that will provide a rigorous evidence- based reference for the clinical use of DSI. However, there are some potential limitations in the proposed study. For example, the dose and course of treatment in different clinical studies cannot be absolutely unified; however, the inconsistency will be reduced by setting subgroups. The study protocol will be executed strictly in accordance with the NMA steps, and has been registered in the PROSPERO registry (CRD42018092705).

## Acknowledgments

We thank Kelly Zammit, BVSc, from Liwen Bianji, Edanz Group China (www.liwenbianji.cn/ac), for editing the English text of a draft of this manuscript.

## Author contributions

The entire manuscript was written and guaranteed by ZZ. The search strategy was formulated by YW and WL. ZZ and HL will complete study selection and data extraction. WL and HL will assess the risk of bias in each study. The final statistical analysis will be completed by ZZ, YW, WL, and HL together. If controversial issues arise in the process, it will be decided by DW. Subsequent versions of this protocol will be reviewed, revised, and approved by all authors.

**Data curation:** Yuanping Wang.

**Funding acquisition:** Dawei Wang.

**Methodology:** Weilin Liao.

**Software:** Huimin Li.

**Writing – original draft:** Zehao Zhu.
